# Clinical and mutational profiles of adult medulloblastoma groups

**DOI:** 10.1186/s40478-020-01066-6

**Published:** 2020-11-10

**Authors:** Gabriel Chun-Hei Wong, Kay Ka-Wai Li, Wei-Wei Wang, Anthony Pak-Yin Liu, Queenie Junqi Huang, Aden Ka-Yin Chan, Manix Fung-Man Poon, Nellie Yuk-Fei Chung, Queenie Hoi-Wing Wong, Hong Chen, Danny Tat Ming Chan, Xian-Zhi Liu, Ying Mao, Zhen-Yu Zhang, Zhi-Feng Shi, Ho-Keung Ng

**Affiliations:** 1 Department of Anatomical and Cellular Pathology, The Chinese University of Hong Kong, Shatin, Hong Kong; 2 Department of Pathology, The First Affiliated Hospital of Zhengzhou University, Zhengzhou, China; 3 Department of Paediatrics and Adolescent Medicine, The University of Hong Kong, Pok Fu Lam, Hong Kong; 4 Department of Pathology, Huashan Hospital, Fudan University, Shanghai, China; 5 Division of Neurosurgery, Department of Surgery, The Chinese University of Hong Kong, Shatin, Hong Kong; 6 Department of Neurosurgery, The First Affiliated Hospital of Zhengzhou University, Zhengzhou, China; 7 Department of Neurosurgery, Huashan Hospital, Fudan University, Shanghai, China

**Keywords:** Adult medulloblastoma, Molecular group, Targeted sequencing, TP53, MYC, KMT2C

## Abstract

Adult medulloblastomas are clinically and molecularly understudied due to their rarity. We performed molecular grouping, targeted sequencing, and TERT promoter Sanger sequencing on a cohort of 99 adult medulloblastomas. SHH made up 50% of the cohort, whereas Group 3 (13%) was present in comparable proportion to WNT (19%) and Group 4 (18%). In contrast to paediatric medulloblastomas, molecular groups had no prognostic impact in our adult cohort (*p* = 0.877). Most frequently mutated genes were TERT (including promoter mutations, mutated in 36% cases), chromatin modifiers KMT2D (31%) and KMT2C (30%), TCF4 (31%), PTCH1 (27%) and DDX3X (24%). Adult WNT patients showed enrichment of TP53 mutations (6/15 WNT cases), and 3/6 TP53-mutant WNT tumours were of large cell/anaplastic histology. Adult SHH medulloblastomas had frequent upstream pathway alterations (PTCH1 and SMO mutations) and few downstream alterations (SUFU mutations, MYCN amplifications). TERT promoter mutations were found in 72% of adult SHH patients, and were restricted to this group. Adult Group 3 tumours lacked hallmark MYC amplifications, but had recurrent mutations in KBTBD4 and NOTCH1. Adult Group 4 tumours harboured recurrent mutations in TCF4 and chromatin modifier genes. Overall, amplifications of MYC and MYCN were rare (3%). Since molecular groups were not prognostic, alternative prognostic markers are needed for adult medulloblastoma. KMT2C mutations were frequently found across molecular groups and were associated with poor survival (*p* = 0.002). Multivariate analysis identified histological type (*p* = 0.026), metastasis (*p* = 0.031) and KMT2C mutational status (*p* = 0.046) as independent prognosticators in our cohort. In summary, we identified distinct clinical and mutational characteristics of adult medulloblastomas that will inform their risk stratification and treatment.

**Electronic supplementary material:**

The online version of this article (10.1186/s40478-020-01066-6) contains supplementary material, which is available to authorized users.

## Introduction

Medulloblastoma is one of the most common malignant brain tumours in children [52]. Medulloblastomas are now classified into four major molecular groups (WNT-activated, SHH-activated, Group 3, and Group 4) with distinct clinical, genetic and transcriptomic profiles [23, 32, 39, 67]. WNT medulloblastoma patients have the best 5-year overall survival rate of over 90%, while Group 3 patients have the worst 5-year overall survival rate of merely 50% [30]. Molecular groups have been incorporated into risk stratification and treatment algorithms of medulloblastoma [22, 48]. For instance, clinical trials are investigating the reduction of irradiation dose to low-risk WNT patients (NCT01878617, NCT02724579, NCT02066220).

In adults, medulloblastomas account for less than 1% of central nervous system (CNS) tumours [52]. Due to their rarity, prospective trials on adult medulloblastomas are limited [40]. The management of adult medulloblastomas is adapted from paediatric protocols, often resulting in dose-limiting toxicities [12].

There is evidence of clinical and genetic differences between adult and paediatric medulloblastomas, suggesting that adult medulloblastomas should be treated and stratified for risk differently [4, 34]. Clinically, adult medulloblastomas more commonly occur in the cerebellar hemispheres [5], are infrequently metastatic at diagnosis [30], and characteristically present with late relapses [3, 10, 52]. Histologically, large cell/anaplastic (LCA) features are less frequently found in adult than in paediatric medulloblastomas [30]. Molecularly, SHH is the predominant group in adult medulloblastomas, while Group 3 is rare [2, 30, 58, 74]. The survival outcomes of molecular groups in adult medulloblastoma have been inconsistent in the literature [30, 58, 74], although some studies suggest that adult WNT patients do not share the excellent survival of paediatric WNT patients [21, 58, 65], and adult SHH patients have relatively favourable survival compared to paediatric SHH patients [6, 65]. Adult medulloblastomas also have distinct cytogenetic profiles from paediatric patients, with chromosome 10q loss and 17q gain proposed as prognostic markers in adults [31, 46].

Despite these initial findings, genome sequencing studies on adult medulloblastomas are still lacking. Knowledge on genetic aberrations in adult medulloblastomas is mostly limited to the SHH group [29, 46]. Comprehensive evaluation of adult medulloblastoma is needed to inform its risk stratification and treatment.

In this study, we report the clinical and mutational profiles of 99 adult medulloblastomas, investigated for molecular group, coding mutations, TERT promoter mutations, MYC and MYCN amplifications, and survival outcome.

## Materials and methods

### Tumour material and patient characteristics

Tumour samples and clinicopathological information were collected from 99 adult medulloblastoma patients between years 2005 and 2018, from the Prince of Wales Hospital (Hong Kong), Huashan Hospital (Shanghai) and the First Affiliated Hospital of Zhengzhou University (Zhengzhou). Local ethics approvals were obtained from The Joint Chinese University of Hong Kong—New Territories East Cluster Clinical Research Ethics Committee, and the Ethics Committees of Huashan Hospital, Shanghai and The First Affiliated Hospital of Zhengzhou University, Zhengzhou. Clinical information was retrieved from institutional paper and electronic records. Survival data was obtained from follow-up clinic visits and direct contact with patients or close relatives via phone.

Haematoxylin and eosin-stained (H&E) slides of all cases were centrally reviewed (H.K.N., A.K.C.) for confirmation of diagnosis and assignment of histological type. All patients were aged > 18 years at the time of diagnosis.

### Molecular group affiliation

The medulloblastomas were assigned to molecular groups by NanoString assay as described by Northcott et al. [50], a transcription-based classification method that is suitable for formalin-fixed paraffin embedded (FFPE) tissues [13, 51]. In brief, RNA was extracted from FFPE tissues using RNeasy FFPE Kit (Qiagen), then quantified by NanoDrop 2000 spectrophotometer (Thermo Scientific). 100 ng RNA per sample was then hybridised to the NanoString nCounter CodeSet at 67 °C for 20 h. The custom CodeSet contained gene-specific probes that assayed the abundance of 22 medulloblastoma group-specific genes and 3 housekeeping genes [50]. Hybridisation complexes were purified with magnetic beads and immobilised on a streptavidin-coated cartridge using the nCounter Prep Station (NanoString Technologies) according to the manufacturer’s protocol. Signals of fluorescent barcodes representing individual target RNA molecules were then counted and recorded by the nCounter Digital Analyzer (NanoString Technologies). Using an R script kindly provided to us by Prof. Michael Taylor, raw data was normalised with R package ‘NanoStringNorm’, and group predictions were made with package ‘pamr’ [50]. NanoString raw counts, expression heatmap and group prediction results can be found in supplementary data (Additional file 1: Figure S1, Additional file 2: Table S2).

### Targeted DNA sequencing, variant and copy number calling

DNA was extracted from FFPE tissues using GeneRead DNA FFPE Kit (Qiagen), then qualified and quantified with QIAseq DNA QuantiMIZE Assay Kit. Targeted next-generation sequencing (NGS) libraries were prepared with a custom QIAseq Targeted DNA Panel, covering the coding exons of 69 genes altered in medulloblastoma and other CNS tumours (Additional file 2: Table S3). The 260-kilobase target region was sequenced with MiSeq v3 (Illumina) to 369.45 × mean coverage per sample (range 99.76–1457.32).

Paired-end reads were aligned to the hg19 (GRCh37) build of the human reference genome with BWA-MEM on GeneGlobe platform (Qiagen). Variants were called using smCounter2 [69] and annotated using wANNOVAR [70]. We excluded variants that did not pass quality filters [69], had variant allele fractions of < 5% or variant allele counts of < 5, or had minor allele frequencies of > 1% in East Asians or the overall human population (as documented in 1000 Genomes, ExAc, gnomAD exome and genome databases). Non-synonymous single nucleotide variants (SNVs) and insertions/deletions (indels) in exonic regions were visualised using Oncoprinter and MutationMapper on cBioPortal [7, 19].

Focal gene-level copy numbers for MYC and MYCN were called using the quandico algorithm [57], with 8 non-tumour brain samples as controls. Amplification was defined as copy number > 10.

### Sanger sequencing for TERT promoter hotspot mutations

A previous whole genome sequencing study identified the TERT promoter as the only non-coding region that is recurrently mutated in medulloblastoma [45]. Accordingly, we performed Sanger sequencing to evaluate the mutational hotspots of TERT promoter, C228T and C250T (124 and 146 bp upstream of the ATG start site respectively), as previously described [1, 8, 9, 37, 64, 71, 72].

Tumour tissues were scraped off FFPE sections, placed in 10 mM Tris–HCl buffer (pH 8.5) with proteinase K, and incubated at 56 °C overnight followed by 98 °C for 10 min. The lysate was then spin down at full speed and the supernatant was collected for subsequent PCR reaction. The 20 μl amplification reaction contained 0.5 μl cell lysate, 0.3 μM forward (5′-GTCCTGCCCCTTCACCTT-3′) and reverse (5′-CAGCGCTGCCTGAAACTC-3′) primers, and 10 μl KAPA HiFi HotStart ReadyMix (Sigma). PCR conditions consisted of 95 °C for 5 min; followed by 45 cycles of 98 °C for 20 s, 68 °C for 15 s, and 72 °C for 30 s; and finally, 72 °C for 1 min, on Veriti 96-Well Thermal Cycler (Applied Biosystems). PCR products were cleaned with spin column-based nucleic acid purification kit (iNtRON Biotechnology) and sequenced with BigDye Terminator Cycle Sequencing kit v1.1 (Life Technologies). The products were resolved in 3130xl Genetic Analyzer (Applied Biosystems). All mutations were confirmed by sequencing of a newly amplified fragment.

### Statistical analysis

Statistical analyses were performed using IBM SPSS Statistics Version 22.

Overall survival (OS) was defined as the time from tumour diagnosis to death or last follow-up. Progression-free survival (PFS) was defined as the time from diagnosis to recurrence or progression as evidenced by radiological imaging, or last follow-up. Univariate analysis was performed on OS using the Kaplan–Meier method and log-rank test. For multivariate analysis, Cox proportional hazards model was applied with OS as the outcome variable. Significance level of α = 0.05 (two-tailed) was used. For multiple comparisons of molecular markers, the Benjamini–Hochberg procedure was employed to control the false discovery rate at Q = 0.05.

## Results

### Clinical characteristics of adult medulloblastomas

**Fig. 1:**
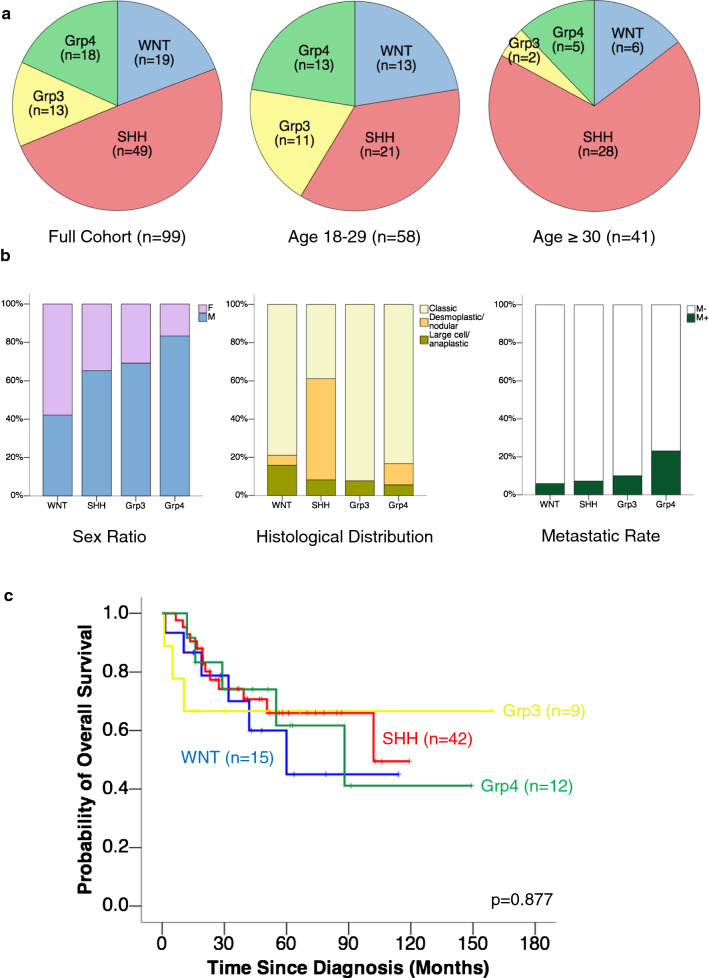
Molecular groups of adult medulloblastomas. **a** The SHH group made up half (49/99) of the cohort. In older adults (age ≥ 30), SHH accounted for 68% (28/41) of cases. Group 3 formed 13% of our adult cohort. **b** The four groups showed differences in sex ratios, histological distributions and metastatic rates. **c** Molecular groups had no prognostic impact (*p* = 0.877) in adult medulloblastomas

Our cohort consisted of 99 adult medulloblastomas aged above 18 at diagnosis. The median age at diagnosis was 27 (range 19–63). There was a trend of decreasing incidence with age in this cohort, with nearly 60% (58/99) of patients between 19 and 29 years (Table 1). Male-to-female ratio was 1.8:1. The tumours were more frequently located in the cerebellar hemispheres than in the vermis (Table 1). Less than 10% (8/82) were metastatic at diagnosis (Fig. 1b). 62% (61/99) of the tumours exhibited classic histology, whereas desmoplastic/nodular and LCA accounted for 29% (29/99) and 9% (9/99) respectively. In terms of treatment, 85% (62/73) of patients achieved gross total resection. 53% (39/73) received both adjuvant chemotherapy and radiotherapy, 30% (22/73) received radiotherapy only, 4% (3/73) received chemotherapy only, and 12% (9/73) received no adjuvant therapy.

**Table 1:** Summary of clinical characteristics of adult medulloblastoma patients

	N	%
Age		
18–23	29	29.3
24–29	29	29.3
30–35	15	15.2
36–41	13	13.1
42–47	7	7.1
48–53	4	4.0
> 53	2	2.0
Sex		
M	64	64.6
F	35	35.4
Location		
Cerebellar hemisphere	34	34.7
Cerebellar vermis	21	21.4
Cerebellum, NOS	11	11.2
Fourth ventricle	21	21.4
Others	11	11.2
Unknown	1	–
Metastasis		
M+	8	9.8
M−	74	90.2
Unknown	17	–
Histological type		
Classic	61	61.6
Desmoplastic/nodular	29	29.3
Large cell/anaplastic	9	9.1
Resection extent		
Gross total resection	62	84.9
Subtotal resection	11	15.1
Unknown	26	–
Adjuvant therapy		
Chemotherapy and radiotherapy	39	53.4
Radiotherapy only	22	30.1
Chemotherapy only	3	4.1
No adjuvant therapy	9	12.3
Unknown	26	–

*NOS* not otherwise specified

We were able to study the survival of 78 patients. The mean and median follow-up were 60.2 and 52.0 months respectively. Median OS and PFS were 102 and 99 months respectively. Among histological types, desmoplastic/nodular tumours had the best outcome whereas LCA had the worst (*p* = 0.027, desmoplastic/nodular vs LCA). The clinical factors with the strongest prognostic impact were metastasis (*p* = 0.005) and adjuvant therapy (*p* < 0.001) (Additional file 1: Figure S2).

### Molecular groups of adult medulloblastomas

As expected, the SHH group comprised half (49/99) of the adult medulloblastomas in our cohort (Fig. 1a). SHH was further enriched in older adults, making up 68% (28/41) of those aged 30 or above. Notably, Group 3 formed 13% (13/99) of our adult cohort (Additional file 1: Figure S3). WNT accounted for 19% (19/99) of cases, and Group 4 accounted for 18% (18/99).

The four molecular groups varied in sex ratio, metastatic rate, and histological distribution (Fig. 1b). WNT was the only group which showed female preponderance, while Group 4 showed the highest male-to-female ratio of 5:1. Group 4 also had the highest metastatic rate among the four groups. Histological type was significantly associated with molecular group (*p* < 0.001, Chi squared test), with 90% (26/29) of desmoplastic/nodular tumours belonging to the SHH group.

Unlike paediatric medulloblastomas, molecular groups had no impact on overall survival in our adult medulloblastoma cohort (*p* = 0.877) (Fig. 1c). The 5-year OS rate of WNT tumours in our cohort was 45%, in contrast to the over 90% 5-year OS rate characteristically attributed to paediatric WNT [30].

### Mutational profiles of adult medulloblastomas

We performed targeted sequencing on 70 cases with sufficient tissues, including 15 WNT, 33 SHH, 10 Group 3, and 12 Group 4 tumours. The mutational load differed significantly between groups (*p* < 0.001, Kruskal–Wallis test): WNT, SHH, Group 3 and Group 4 each recorded a median of 7, 6, 3 and 2.5 mutations per case respectively.

**Fig. 2:**
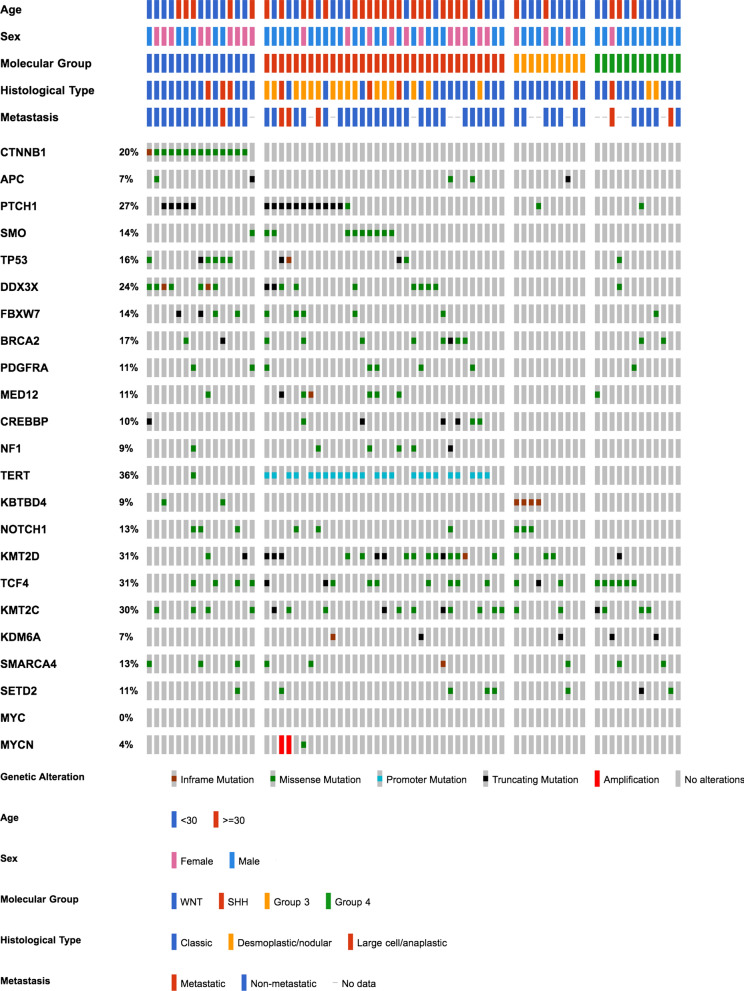
Oncoprint summary of clinical and mutational profiles of 70 sequenced adult medulloblastomas according to molecular group

The most frequently mutated genes were TERT (including promoter mutations, mutated in 36% of cases which underwent NGS), KMT2D (31%), TCF4 (31%), KMT2C (30%), PTCH1 (27%) and DDX3X (24%) (Fig. 2). Among these, TERT promoter mutations were restricted to the SHH group, while PTCH1 and DDX3X mutations were mostly found in WNT and SHH. KMT2C, KMT2D and TCF4 mutations were seen across all four groups.

Amplification of MYC was not found throughout the adult cohort, while two SHH cases showed high-level MYCN amplifications (Fig. 2, Additional file 2: Table S6).

#### WNT group

14/15 of the sequenced WNT cases carried hotspot mutations in CTNNB1, all concentrated in amino acid positions D32–S37 (particularly S33) (Additional file 1: Figure S4a). 2 cases were mutated in APC. Interestingly, canonical SHH pathway genes were also altered in the WNT group: 5 WNT cases harboured PTCH1 truncating mutations that co-occurred with CTNNB1 mutations, and another case showed mutations in both APC and SMO (Additional file 1: Figure S5).

**Fig. 3:**
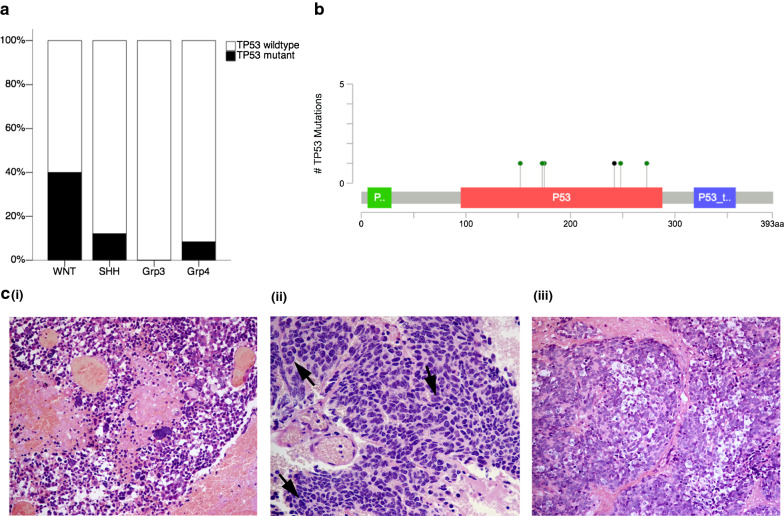
Unique occurrence of TP53-mutant large cell/anaplastic WNT medulloblastomas. **a** TP53 mutations were enriched in adult WNT medulloblastomas, found in 40% (6/15) of cases in this group. **b** All TP53 mutations in WNT occurred within the p53 DNA binding domain. **c** 3/6 TP53-mutant adult WNT cases were of large cell/anaplastic histology; (i) 19/F, with missense mutation TP53 V173A; (ii) 20/M, with missense mutation TP53 R175H; (iii) 32/F, with missense mutation TP53 R248W; arrows show nuclear moulding

Mutations in TP53 were found in 40% (6/15) of our adult WNT cases (Fig. 3a), a much higher proportion than the 13–16% reported in WNT tumours within paediatric-predominant cohorts [45, 47, 75]. All TP53 mutations in WNT occurred within the p53 DNA binding domain (Fig. 3b). Half (3/6) of the TP53-mutant WNT tumours showed LCA histology (Fig. 3c), while all of the TP53-wildtype WNT tumours displayed classic histology.

Other frequently mutated genes in WNT included DDX3X (47%) and FBXW7 (27%). Mutations in DDX3X concentrated in its two helicase domains (Additional file 1: Figure S4d), whereas an R385C/H hotspot was identified in FBXW7 (Additional file 1: Figure S4e).

#### SHH group

Among the SHH pathway genes, PTCH1 was mutated in 36% (12/33) of adult SHH medulloblastomas, and SMO was mutated in 27% (9/33) (Fig. 2). Most (11/12) PTCH1 mutations were truncating (Additional file 1: Figure S4f), whereas SMO mutations mainly (8/9) consisted of a hotspot substitution L412F (Additional file 1: Figure S4g). No SUFU mutation was found in adult SHH, consistent with previous studies [29, 45].

TERT promoter mutations were limited to the SHH group, detected in 72% (34/47) of adult SHH cases. C228T was found in 28 cases, whereas C250T was found in 4; one case showed C228A and another showed C250G (Additional file 1: Figure S6, Additional file 2: Table S5).

Other prevalently seen mutations in adult SHH included those of DDX3X (27%), BRCA2 (24%), MED12 (18%), CREBBP (18%), FBXW7 (15%), PDGFRA (15%) and NF1 (15%). DDX3X, CREBBP and FBXW7 mutations were reported to be very rare in paediatric SHH [29, 45]. TP53 mutations were rarer than in WNT, present in 12% (4/33) of SHH cases.

MYCN amplification was found in 2 SHH cases. The 2 cases exhibited high-level amplifications, with 123 and 78 copies respectively (Additional file 2: Table S6). Both of these cases had concomitant TP53 mutation and were metastatic (Fig. 2).

#### Group 3

Hotspot in-frame insertions of KBTBD4 were found in 40% (4/10) of Group 3 cases (Additional file 1: Figure S4h). NOTCH1, KMT2D and TCF4 were each mutated in 3 cases. Of these, all NOTCH1 mutations co-occurred with KBTBD4 insertions.

MYC amplification, a hallmark high-risk feature almost exclusive to Group 3 [45, 47, 49], was absent in our adult cohort, including in Group 3 tumours (Fig. 2).

#### Group 4

TCF4 mutations were found in half (6/12) of the Group 4 cases. All 6 mutations consisted of a missense substitution Q95R, a hotspot that was identified across groups (Additional file 1: Figure S4j). Other recurrently mutated genes in Group 4 included chromatin modifiers KMT2C (4/12), KDM6A (2/12), SETD2 (2/12) and SMARCA4 (2/12).

No Group 4 cases harboured MYCN amplification (Fig. 2).

#### Chromatin modification genes

Overall, mutations in genes related to chromatin modification were found in 81% (57/70) of samples, distributed across all four groups (Additional file 1: Figure S7). These genes included histone modifiers and their interacting proteins (KMT2D, KMT2C, KDM6A, SETD2, CREBBP, BCOR, GSE1, ZMYM3), SWI/SNF-nucleosome remodelling complex subunits (SMARCA4, SMARCB1, ARID1A, ARID2), as well as histones (H3F3A) and their chaperones (ATRX).

### Prognostication of adult medulloblastomas

**Fig. 4:**
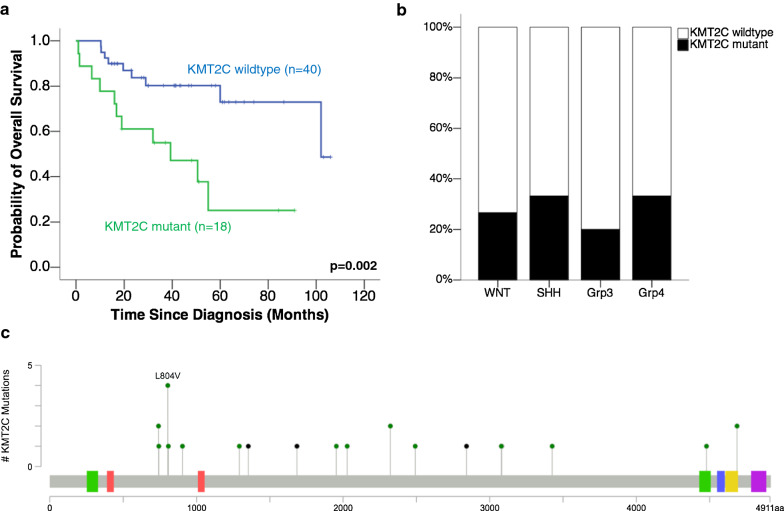
KMT2C mutations as a poor prognostic marker in adult medulloblastomas. **a** KMT2C mutation was associated with poor overall survival (*p* = 0.002). **b** KMT2C mutations were found in 30% of adult medulloblastomas across molecular groups. **c** KMT2C mutations were scattered across the coding region of the gene

Since molecular groups had no impact on the overall survival of adult medulloblastoma patients (Fig. 1c), we assessed the prognostic significance of frequently mutated genes in our cohort. Presence of KMT2C mutation was associated with poor outcome (*p* = 0.002, *q* = 0.034) (Table 2, Fig. 4a). KMT2C mutations were present in 30% of adult medulloblastoma cases, distributed across all four molecular groups (Fig. 4b). At the gene level, mutations were scattered across the coding region of KMT2C (Fig. 4c).

**Table 2:** Univariate analysis for mutational statuses of genes mutated in ≥ 10% cases in adult medulloblastoma cohort

Gene	Mutational frequency (%)	OS log-rank *p* value	Benjamini–Hochberg *q* value
TERT promoter	39^a^	0.294	0.460
KMT2D	31	0.056	0.334
TCF4	31	0.282	0.460
KMT2C	30	**0.002**	**0.034**
PTCH1	27	0.356	0.460
DDX3X	24	0.969	0.969
CTNNB1	20	0.565	0.640
BRCA2	17	0.337	0.460
TP53	16	0.062	0.334
FBXW7	14	0.118	0.334
SMO	14	0.101	0.334
NOTCH1	13	0.379	0.460
SMARCA4	13	0.172	0.418
PDGFRA	11	0.873	0.928
MED12	11	0.102	0.334
SETD2	11	0.257	0.460
CREBBP	10	0.314	0.460

Bold values are *p*<0.05 and *q*<0.05

^a^34/88 cases by Sanger sequencing

Upon multivariate analysis, KMT2C mutation remained as an independent poor prognosticator (hazard ratio (HR) = 6.468, *p* = 0.046) after adjusting for age, sex, molecular group, histological type, metastasis and adjuvant therapy (Table 3). Other independent prognosticators included histological type (*p* = 0.026) and metastasis (*p* = 0.031). Molecular groups continued to show no prognostic impact in the multivariate model (*p* = 0.407).

**Table 3:** Multivariate analysis of clinical and molecular prognosticators in adult medulloblastomas

	HR (95% CI)	*p* value
Age	0.869 (0.755–1.002)	0.053
Sex (male versus female)	27.878 (0.382–2035.342)	0.128
Molecular group		0.407
SHH versus WNT	0.894 (0.022–36.018)	0.953
Group 3 versus WNT	5.903 (0.225–155.063)	0.287
Group 4 versus WNT	0.306 (0.006–14.994)	0.551
Histological type		**0.026**
Desmoplastic/nodular versus classic	0.467 (0.046–4.746)	0.520
LCA versus classic	129.534 (2.871–5844.831)	**0.012**
Metastasis	7.78 (1.207–50.147)	**0.031**
Adjuvant therapy	0.283 (0.007–11.612)	0.506
KMT2C mutation	6.468 (1.035–40.404)	**0.046**

Bold values are *p*<0.05 and *q*<0.05

## Discussion

In this study, we showed that molecular groups have no prognostic significance in adult medulloblastomas. This is in contrast to paediatric medulloblastomas where molecular groups have been integrated into risk stratification schemes [55, 56]. In particular, WNT status was not associated with favourable survival in our adult cohort, in agreement with a previous study by Korshunov et al. [31]. With the increased interest in the feasibility of reducing irradiation dose to WNT patients [44], caution should be taken in applying such treatment de-escalation approaches to adult WNT patients.

When examining the mutational profiles of adult WNT medulloblastomas, we discovered a high frequency of TP53 mutations, compared to paediatric WNT. TP53 mutations have been reported in 13–16% of WNT medulloblastomas [45, 47, 75], whereas in our adult cohort, TP53 mutations were detected in 40% of WNT cases. Re-analysis of data from Northcott et al. gave a similar result, where 2/4 adult WNT tumours harboured TP53 mutations, compared to only 3/29 paediatric WNT tumours in their cohort [45]. TP53 has been shown to play a role in WNT pathophysiology: excess β-catenin promotes accumulation of transcriptionally active p53 [14], and activated p53 in turn downregulates β-catenin [35, 62], indicating that p53 mediates an important tumour suppressive mechanism against WNT pathway activation. Gibson et al. showed that concomitant TP53 deletion was required to induce medulloblastoma formation in CTNNB1-mutant mice [20]. The abundance of TP53 mutations in adult WNT may partly account for the biological and clinical differences observed between adult and paediatric WNT tumours.

Apart from the significant enrichment of TP53 mutations in adult WNT, we also observed that a high proportion of TP53-mutant adult WNT tumours shared the high-risk feature of LCA histology. This is in contrast to the mostly paediatric cohort in Zhukova et al., where none of the TP53-mutant WNT tumours showed anaplastic features [75]. The unique occurrence of TP53-mutant LCA WNT tumours, and the heterogeneous treatments received [44], may be reasons for the lack of favourable survival for WNT patients in our adult cohort.

Another striking feature of adult WNT medulloblastomas is the concurrent mutations of WNT and SHH pathway genes. This coincides with the recent observations by Iorgulescu et al., who found SHH pathway mutations at subclonal allele frequencies in 3/7 of their CTNNB1-mutant medulloblastomas [25]. They subsequently performed immunohistochemistry for GAB1, which yielded a focal staining pattern that confirmed secondary SHH pathway activation, reflecting intratumoural heterogeneity within these WNT medulloblastomas. Medulloblastomas have been shown to exhibit substantial spatial heterogeneity in genetic alterations, which points toward the need for multi-regional biopsies and combination targeted therapies [43].

SHH is the predominant group in adult medulloblastomas, and adult SHH tumours are characterised by upstream pathway mutations in PTCH1 and SMO, whereas downstream pathway alterations such as SUFU mutations and MYCN amplifications are rare in this age group. Our findings are similar to those of Kool et al., who also found that a large proportion of adult SHH tumours are targetable by the SMO inhibitor LDE-225 (sonidegib), due to the rarity of SHH pathway alterations downstream to SMO which confer therapeutic resistance [29]. A phase II trial showed clinical efficacy of the SMO inhibitor vismodegib in adult recurrent SHH medulloblastoma [60].

The strong enrichment of TERT promoter mutations in adult SHH medulloblastomas has been reported by multiple studies [28, 29, 38, 59]. In addition to the TERT promoter, our study confirmed that gene mutations in DDX3X, CREBBP and FBXW7, which are rare in paediatric SHH [29, 45], occur frequently in adult SHH; on the other hand, TP53 mutations which are abundant in paediatric SHH are rarely seen in adults. In 2017, Cavalli et al. further classified SHH medulloblastomas into four age-associated subtypes based on integrated methylation and expression profiling data [6]. Most adult SHH cases belonged to the SHH-δ subtype which was highly enriched for TERT promoter mutations and had relatively favourable survival, further substantiating the hypothesis that adult SHH tumours represent a biologically disparate entity from paediatric and infant SHH tumours.

While previous studies found that Group 3 is extremely rare or absent in adult medulloblastomas [30, 58, 74], our cohort showed that Group 3 could make up a significant proportion of adult medulloblastomas, and that adult Group 3 patients did not have worse outcome than the other groups. We also showed that MYC amplification, the hallmark driver event detected in 12–17% of Group 3 medulloblastomas [45, 47, 49], was absent in adult Group 3 tumours. MYC amplification is a well-established poor prognosticator in various risk stratification models [15, 16, 55, 61, 65], thus the lack of this group-specific marker in adult medulloblastoma might explain why Group 3 patients did not exhibit worse survival than the other groups in our adult cohort.

We also identified other genetic events in adult Group 3, such as KBTBD4 hotspot insertions described earlier by Northcott et al. [45], as well as NOTCH1 mutations which are rare in paediatric Group 3. Kahn et al. recently reported that NOTCH1 signaling regulates the initiation of metastasis and self-renewal of Group 3 medulloblastoma, and intrathecal treatment with a NOTCH1 blocking antibody reduced spinal metastasis and improved survival in vivo [27]. These findings propose NOTCH1 signaling as a potential driver and therapeutic target in Group 3, alongside MYC activation and KBTBD4 insertions [42].

TCF4 mutations were a frequent event in our adult Group 4 medulloblastomas. TCF4 was also one of the most frequently mutated genes in our whole cohort. TCF4 is a transcription factor involved in neurological development and is mutated in 2% of medulloblastomas [45]. Re-analysis of sequencing data from Northcott et al. revealed that TCF4 mutations were enriched in adults, present in 17% (10/58) of adult cases. Whether TCF4 mutations play any functional role in medulloblastoma remains a topic for further investigation.

Lastly, the lack of prognostic impact of molecular groups warrants the discovery of alternative prognostic markers in adult medulloblastoma. In addition to histological type and metastasis, we identified KMT2C mutational status as an independent prognosticator in our cohort. KMT2C, also known as MLL3, is a histone lysine methyltransferase that catalyses the monomethylation of histone H3 lysine 4 (H3K4me) at gene enhancers [24]. KMT2C has a tumour suppressive role across many cancer types [66], and mutations or low expression of KMT2C are associated with poor survival in a wide range of lung, breast, gastric, skin and brain cancers [11, 17, 18, 33, 36, 41, 54, 63, 68, 73]. KMT2C was among the first few recurrently mutated genes identified in early medulloblastoma sequencing studies [53]. In our adult medulloblastoma cohort, KMT2C was one of the most frequently mutated genes, with mutations detected in 30% of cases across ages, sexes, histological types and molecular groups, so it is a potential biomarker for stratifying adult medulloblastoma patients. Our findings reaffirm the central importance of chromatin modification in medulloblastoma pathophysiology [26], and highlight the need for more comprehensive evaluation of the epigenetic landscape of adult medulloblastomas.

## Supplementary information

**Additional file 1.** Supplementary Figures S1–S7

**Additional file 2.** Supplementary Tables S1–S6
