# Supplementary material for: Clinical and mutational profiles of adult medulloblastoma groups
Source: Acta Neuropathol Commun. 2020 Nov 10;8:191. doi: 10.1186/s40478-020-01066-6 (PMC7656770; doi:10.1186/s40478-020-01066-6)

Supplementary Figure 1. Expression heatmap of 22 medulloblastoma group-specific genes in the NanoString CodeSet across a cohort of 99 adult medulloblastomas

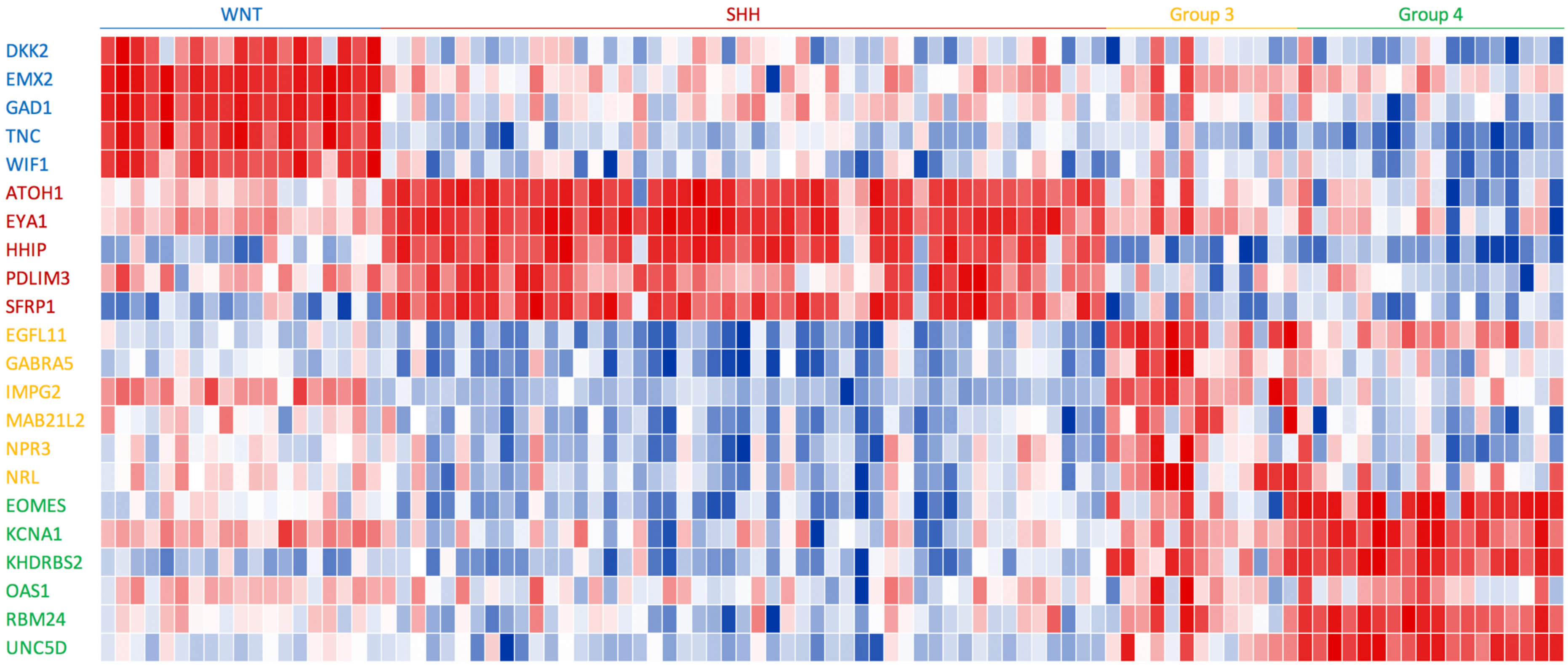

Supplementary Figure 2. Kaplan-Meier survival curves (OS) of adult medulloblastoma patients **(a)**, stratified by **(b)** age group, **(c)** sex, **(d)** location, **(e)** metastasis, **(f)** histological type, **(g)** resection extent and **(h)** adjuvant therapy

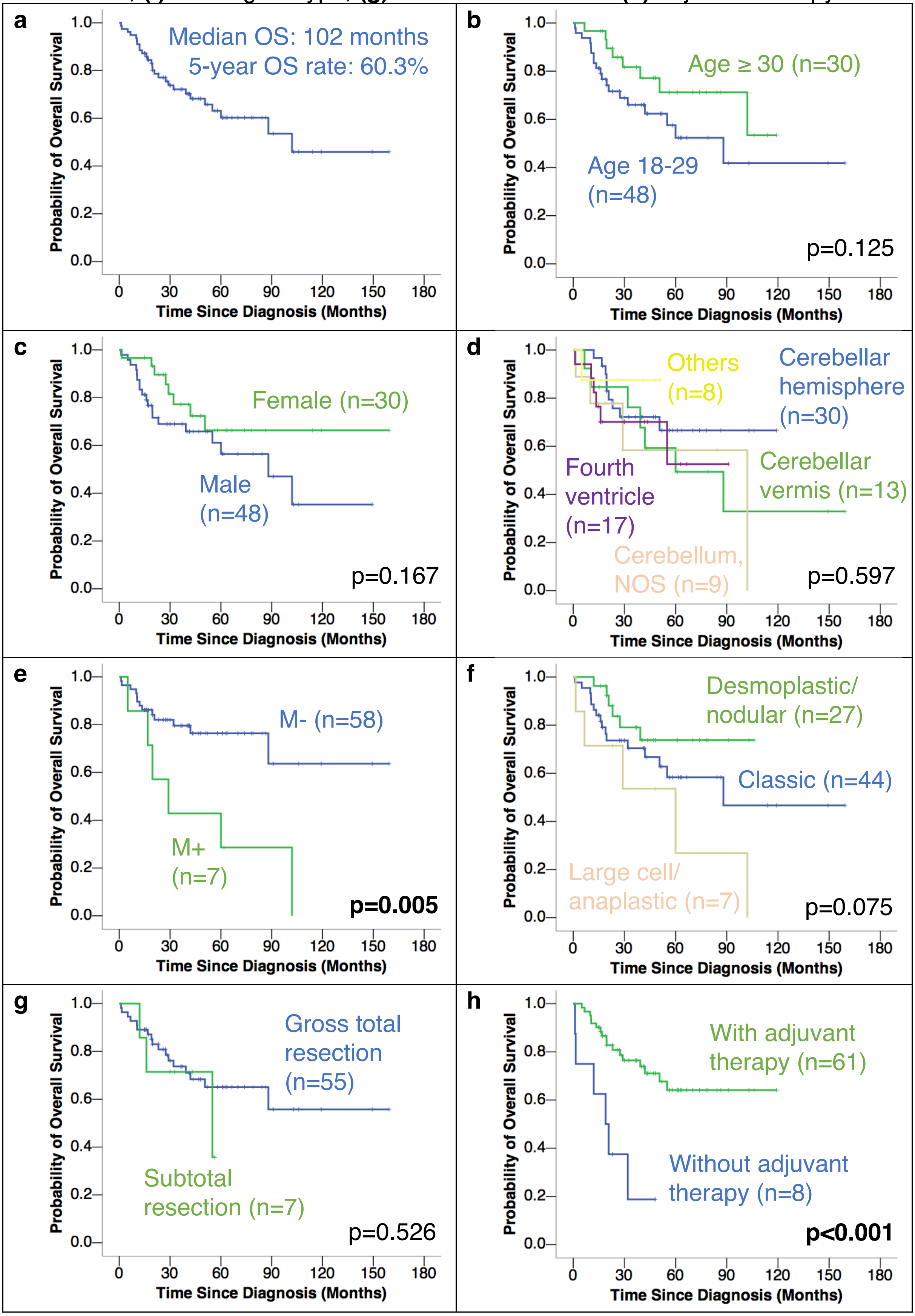

Supplementary Figure 3. H&E photomicrographs of adult Group 3 medulloblastomas

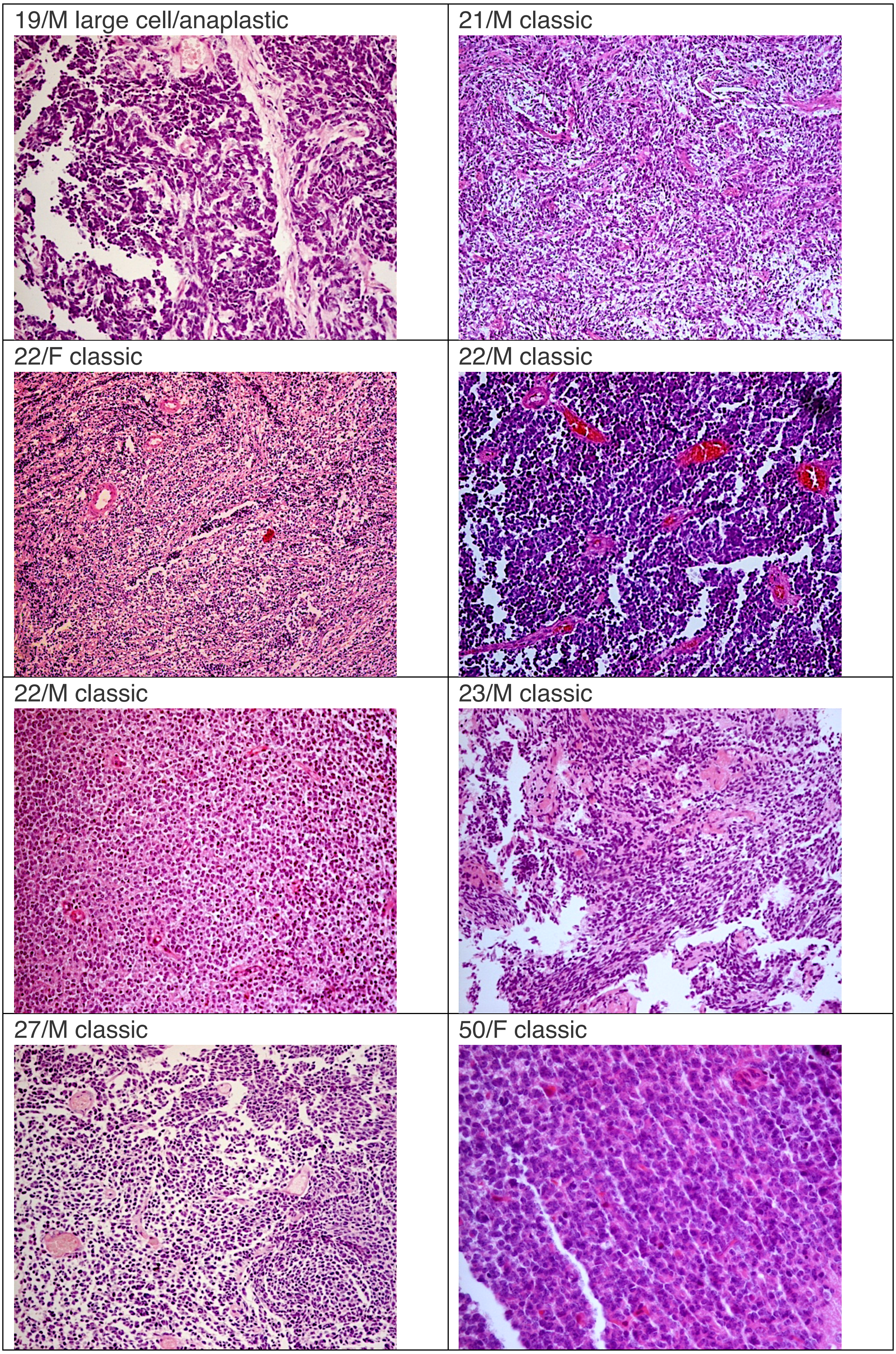

Supplementary Figure 4. Gene-level summary plots of mutations in **(a)** CTNNB1, **(b)** APC, **(c)** TP53, **(d)** DDX3X, **(e)** FBXW7, **(f)** PTCH1, **(g)** SMO, **(h)** KBTBD4, **(i)** NOTCH1 and **(j)** TCF4. Green, missense mutations; Brown: in-frame mutations; Black: truncating mutations

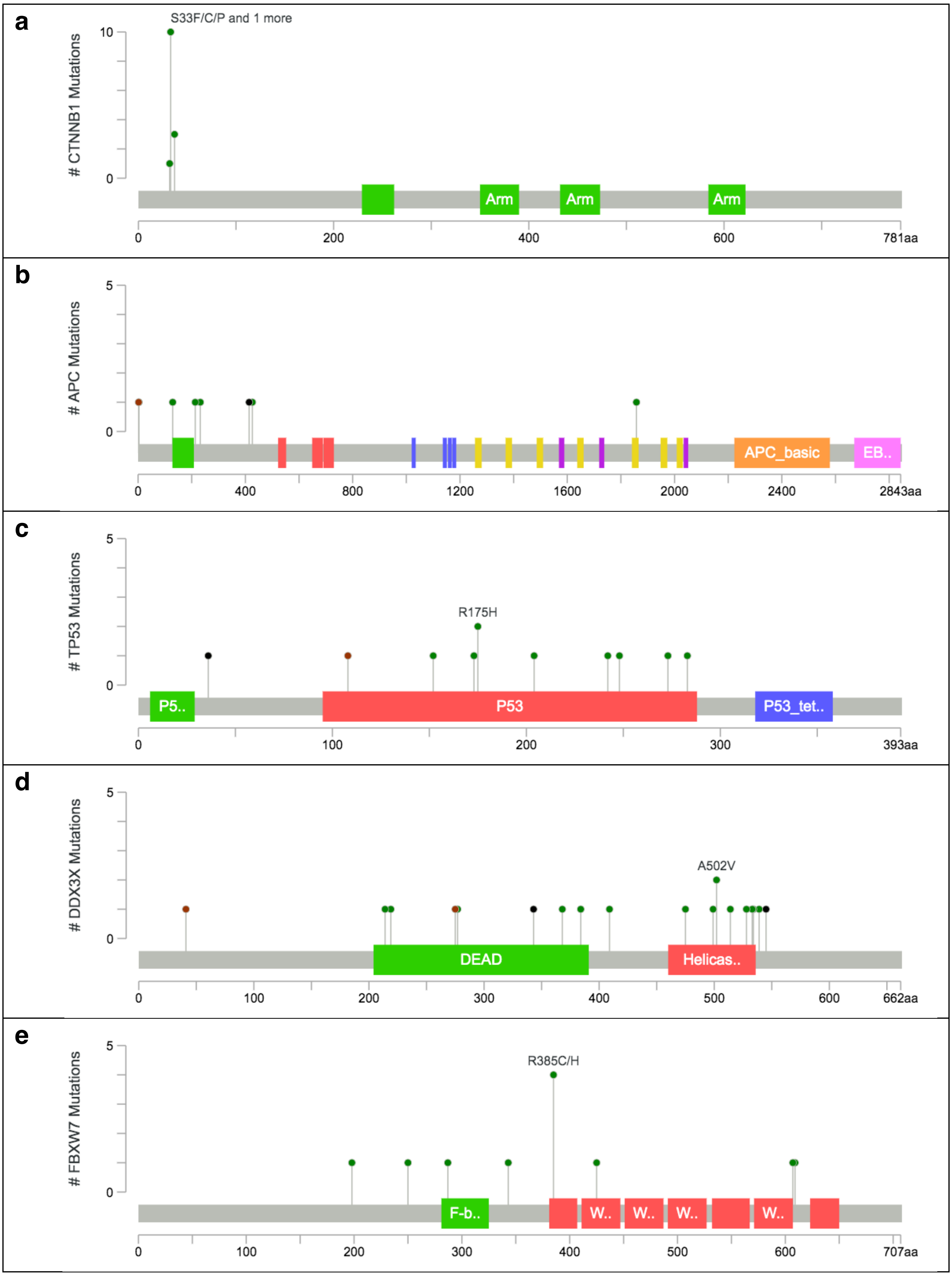

Supplementary Figure 4 (continued). Gene-level summary plots of mutations in **(a)** CTNNB1, **(b)** APC, **(c)** TP53, **(d)** DDX3X, **(e)** FBXW7, **(f)** PTCH1, **(g)** SMO, **(h)** KBTBD4, **(i)** NOTCH1 and **(j)** TCF4. Green, missense mutations; Brown: in-frame mutations; Black: truncating mutations

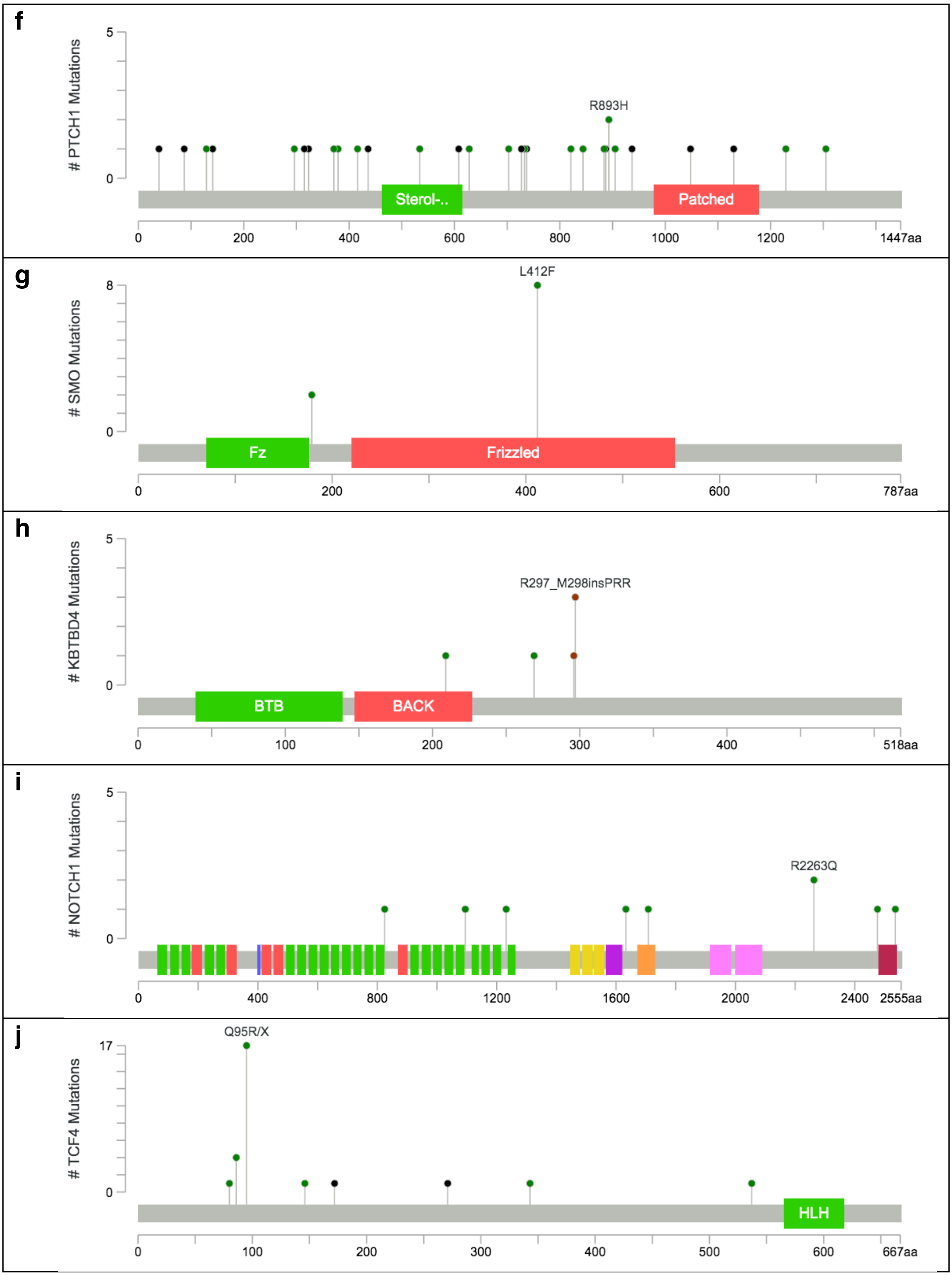

Supplementary Figure 5. Sanger sequencing chromatograms showing concurrent WNT and SHH pathway gene mutations in adult WNT medulloblastomas

Example	Sanger sequencing chromatograms	
43/M, with concurrent CTNNB1 and PTCH1 mutations	CTNNB1<br>NM_001098209<br>c.C98G<br>p.S33C	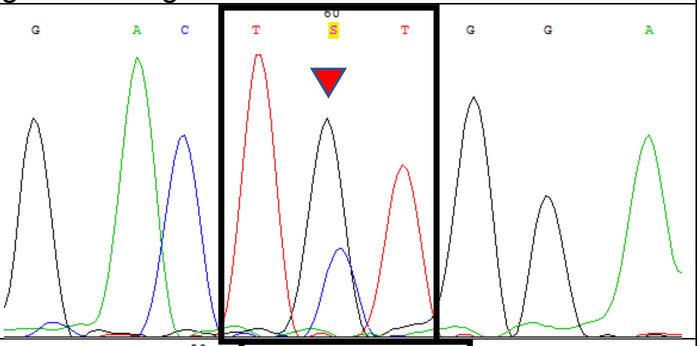
	PTCH1<br>NM_000264<br>c.968delT<br>p.L323fs*0	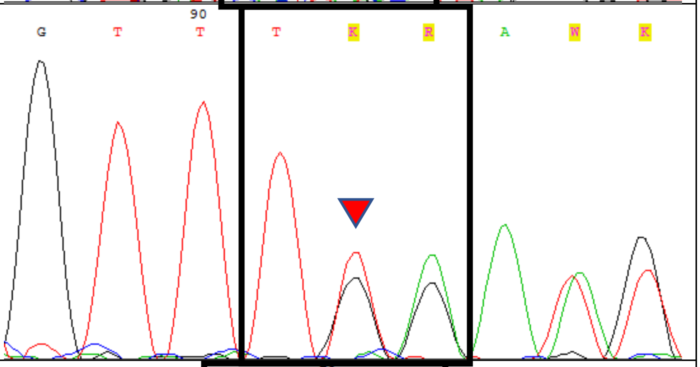
47/F, with concurrent APC and SMO mutations	APC<br>NM_001127511<br>c.G7A<br>p.A3T	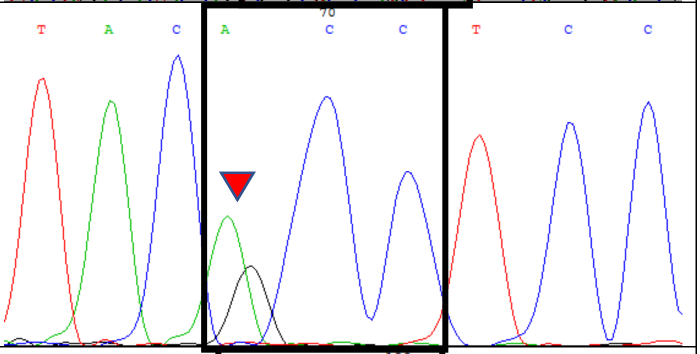
	SMO<br>NM_005631<br>c.C536T<br>p.T179M	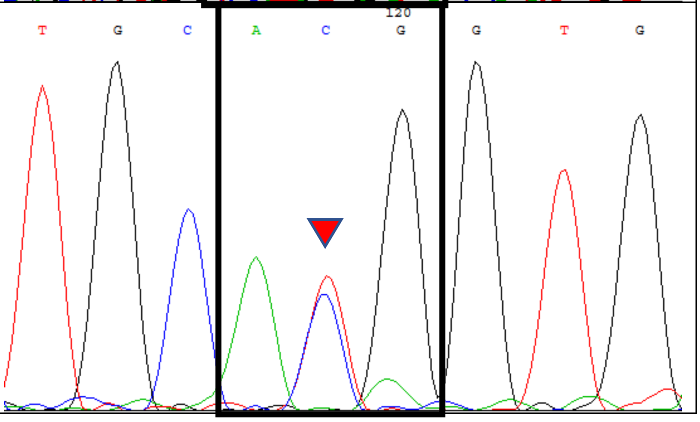

Supplementary Figure 6. Sanger sequencing chromatograms showing TERT promoter mutations in adult SHH medulloblastomas

Genotype (count of SHH cases)	Representative Sanger sequencing chromatogram<br><br>Mutational hotspots:<br>1295250 (-146 from transcription start site)<br>1295228 (-124 from TSS)
Wildtype (13)	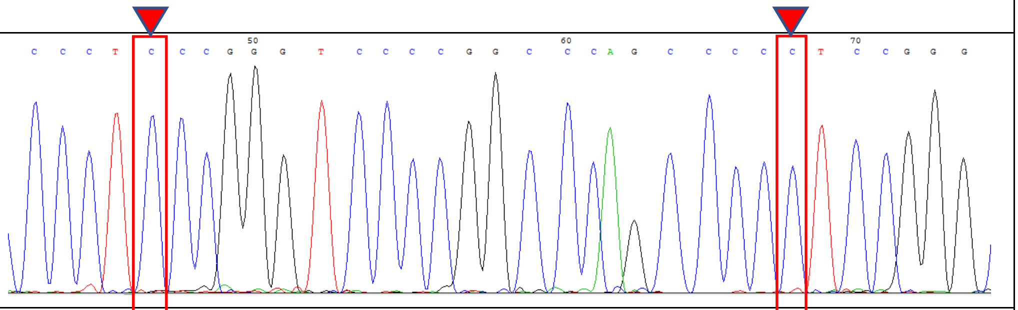
C228T (28)	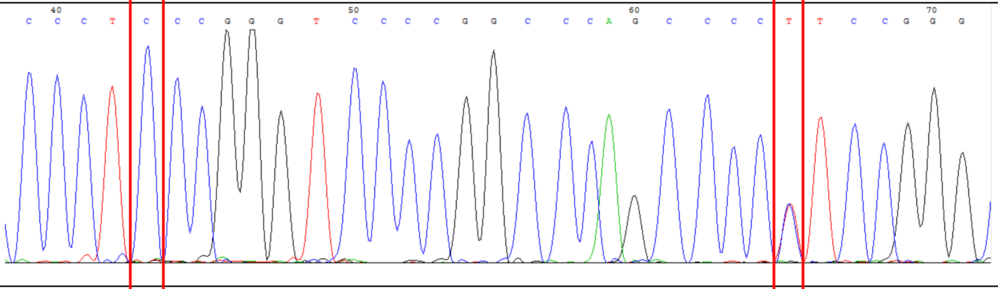
C250T (4)	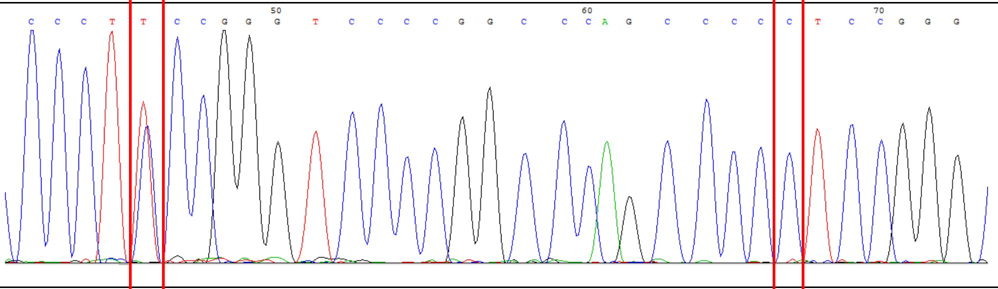
C228A (1)	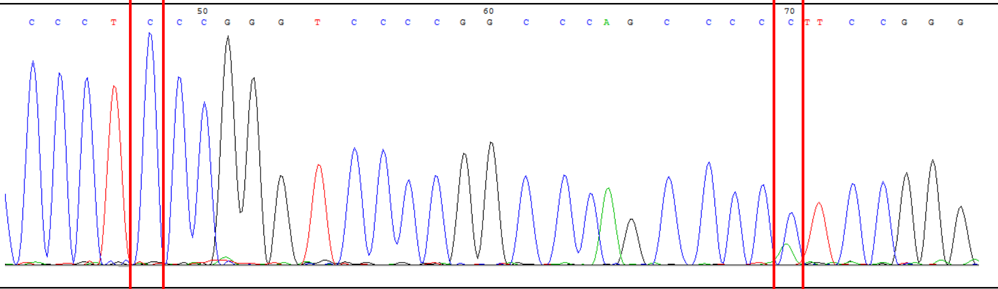
C250G (1)	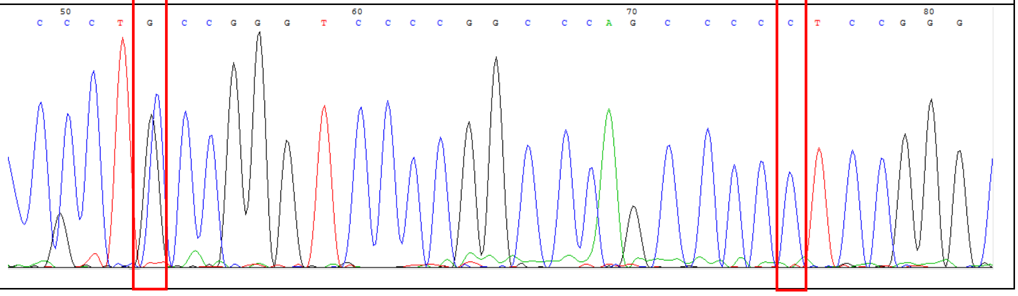

Supplementary Figure 7. Oncoprint summary showing mutations in genes related to chromatin modification

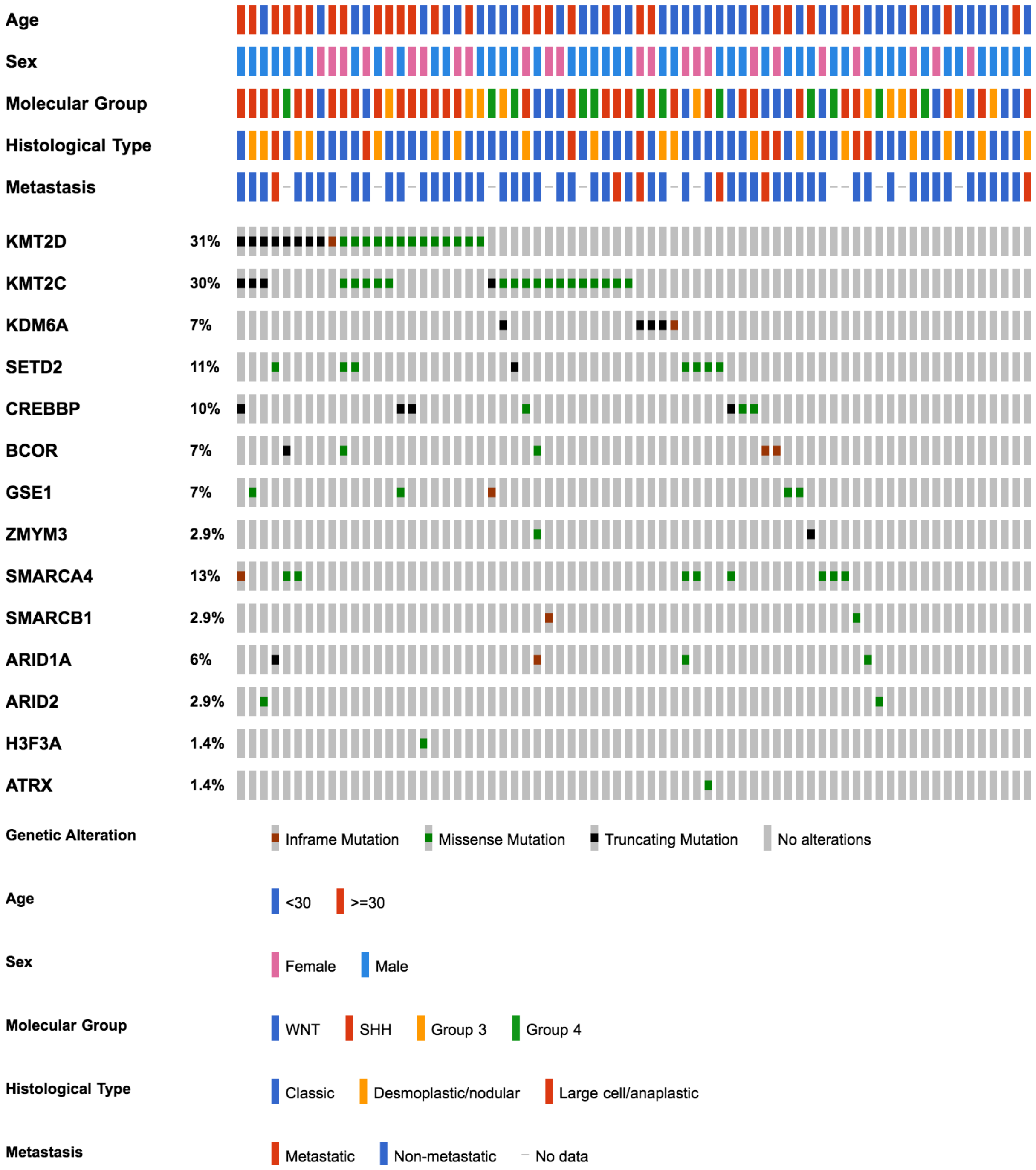

Supplement: Supplementary file 1 — Additional file 1. Supplementary Figures S1–S7 [file 40478_2020_1066_MOESM1_ESM.pdf]
